# Exploring the Opportunities and Challenges of Healthcare Innovation in UK Higher Education: A Narrative Review

**DOI:** 10.3390/nursrep15050171

**Published:** 2025-05-14

**Authors:** Renske Emicke, Ashley Shepherd, Dylan Powell

**Affiliations:** 1 Nursing, Social Work and Therapy, University of Applied Sciences Appollon, 28359 Bremen, Germany; 2 Faculty of Health Sciences and Sport, University of Stirling, Stirling FK9 4LA, UK; ashley.shepherd@stir.ac.uk

**Keywords:** healthcare, innovation, technology transfer, nursing education, higher education, health information technology

## Abstract

**Background**: The healthcare sector is under increasing pressure due to an ageing population, rising multimorbidity, and a projected global workforce shortage of 10 million by 2030. It is becoming increasingly apparent that addressing these challenges requires more than simply increasing workforce numbers—it necessitates a shift towards innovative practices in healthcare education. Higher education (HE) plays a crucial role in preparing future healthcare professionals, yet embedding innovation within HE presents challenges such as resistance to change, resource limitations, and difficulties in interdisciplinary collaboration. This review explores the barriers and opportunities associated with fostering innovation in HE health programmes. **Methods**: This narrative review explored the recent literature on innovation in healthcare HE, examining national policies, institutional strategies, and emerging technological advancements. It describes the impact of digital learning tools, simulation-based education, artificial intelligence (AI), and interprofessional education (IPE) on student learning outcomes and workforce preparedness. **Results**: Findings suggest that while digitalisation and AI-driven innovations offer opportunities to enhance HE health programmes, their effectiveness is dependent on appropriate pedagogical integration and resource. Challenges include upskilling workforce to new more modern methods, ensuring equitable access to digital resources, and maintaining a balance between technological innovation and traditional face-to-face learning. **Conclusions**: To embed innovation effectively within healthcare HE, institutions may need to prioritise interdisciplinary collaboration, entrepreneurial thinking, and sustainable funding models. While technology is key to modernising education, it should be implemented alongside evidence-based pedagogical strategies to ensure meaningful learning outcomes and long-term workforce preparedness.

## 1. Introduction

The healthcare sector faces mounting pressures. Driven by an increasingly ageing and multimorbid population, the complexity of care continues to grow. With a projected global health workforce shortage of 10 million workers by 2030 [1,2], there is an urgent need to explore and implement innovative solutions that can address these challenges effectively. However, to meet these demands in parallel, the transformation of healthcare requires more than an increase in workforce numbers [1]. It necessitates a paradigm shift toward adopting innovative practices tailored to evolving healthcare needs. However, embedding such practices within both clinical and educational settings carries several challenges, including resistance to change and the difficulty of fostering environments conducive to innovation [3]. This underscores the importance of understanding strategies that can facilitate the adoption and integration of innovation effectively. Higher education (HE) plays an important role in the training, education, and research of many health professions. As such, promoting innovation in HE health programmes is pivotal to preparing future healthcare professionals to address these challenges. Establishing a shared understanding of innovation and recognising the barriers that organisations and individuals face when confronted with change are essential steps in this process [3,4]. Innovation is inherently complex, resource-intensive, and often met with hesitance from stakeholders [3,4]. Furthermore, it is a multifaceted phenomenon that varies in scale and scope [5].

This narrative review describes the recent literature to explore the challenges and opportunities associated with implementing innovation within higher education. It aims to provide insights into how innovation can be fostered effectively, addressing resistance, and leveraging educational environments to support change.

## 2. Methods

To address this complex topic, a narrative review of the recent literature was conducted to explore the challenges and opportunities associated with implementing innovation in HE. The search process was conducted in October 2023 using PUBMED. A search was performed using a Boolean search strategy, combining the keywords “Barriers” AND “Innovation” AND “Higher Education”, along with relevant synonyms. Given the complexity of the topic, the inclusion criteria were peer-reviewed publications from 2020 onward, available in German, Dutch, or English, with full-text accessibility, and relevant to the fields of healthcare and education. To identify additional relevant studies, a hand search was conducted to locate materials not retrieved through the systematic search.

Duplicate studies were then removed, and the titles and abstracts of the identified studies were reviewed. Articles that did not align with the topic of interest were excluded. No software was used to sort the available articles. To resolve any uncertainty, the inclusion or exclusion of articles was confirmed through discussions among different authors.

A total of 13 articles were identified. Among these, three did not meet the inclusion criteria, and one lacked full-text availability, resulting in nine articles being included. Five additional studies were identified through the supplementary hand search and assessed as meeting the inclusion criteria, resulting in a total of fourteen included studies. Content analysis was performed using Excel, where descriptive codes were assigned. In the next step, these codes were categorised into themes, guiding the narrative of this article.

## 3. Background to Innovation in Higher Education

### 3.1. Definition of Innovation

Innovation in healthcare lacks a universally agreed-upon definition. Kelly and Young [6] attempt to define it as the combination of “invention + adoption + diffusion” (p. 121), emphasising both the creation and dissemination of new ideas. Similarly, the World Health Organization [7] describes health innovation as something either new or improved. At its core, innovation involves change, leading to ongoing debates about whether change itself is the fundamental element of innovation [8]. Given these discussions, it is essential to explore the challenges faced by both organisations and individuals when encountering change, as there is often resistance to adopting new practices.

In the context of healthcare and rapidly advancing technology, innovation must encompass the application of novel concepts to address the evolving demands of the field [9]. However, innovation is not solely dependent on large-scale advancements. Flessa and Huebner argue that innovation is a process that can vary in scale, ranging from small improvements to transformative changes [5]. Grounding the concept in healthcare, Kaya et al. [4] define innovation as “activities motivated by the desire to improve healthy person/patient care outcomes and the need to reduce costs” (p. 1674).

The COVID-19 pandemic has further highlighted the role of innovation in healthcare, with emerging research assessing its impact and effectiveness. In this context, innovation has been defined as the introduction of new methods, approaches, or processes aimed at improving public health, as well as the implementation of novel ideas that lead to meaningful change [10].

Although the literature provides varying perspectives, an initial analysis suggests that innovation in healthcare is commonly understood as “change” or “something new” in relation to methods, processes, and approaches. However, Pusic and Ellaway make an important distinction, arguing that innovation should be seen as doing something new, while change refers to doing something differently [11]. As efforts to foster innovation in healthcare HE continue, it is crucial to establish a shared understanding of what innovation entails [4,12]. This shared definition could serve as a foundation for adapting HE curricula to ensure that future healthcare professionals are equipped to meet the evolving needs of the field. However, Leary et al. caution against the dilution of the term “innovation” within health education programmes [13], further illustrating the need for further research, analysis, and harmonisation in approaches.

### 3.2. Current State in the United Kingdom

According to the 2023 Academic Ranking of World Universities, the United Kingdom (UK) is home to two universities ranked in the global top ten, underscoring its status as a leading destination for HE. In Scotland, the government has committed to supporting innovation, learning, and entrepreneurship in universities [14]. The Scottish National Innovation Strategy articulates Scotland’s ambition to become “one of the most innovative small nations in the world” (p. 2). This aligns with the national strategy for the health and social care workforce, which aims to adapt the healthcare system to evolving needs [14]. These national strategies reflect a broader trend towards prioritising innovation within HE, which is critical for addressing the healthcare workforce challenges outlined in the introduction.

While there is no universally agreed-upon definition of innovation in higher education, it remains a central theme globally, with various concepts being actively explored. This makes consensus and comparisons difficult. Researchers argue that teaching practices must be grounded in innovative methodologies, fostering the development of competencies and problem-solving skills through cooperative work, critical thinking, and technological support [15]. Research has long advocated for the integration of social innovation activities in HE; however, they acknowledge the lack of understanding and political support for this aspect [16]. These perspectives align with the need for institutions to not only adapt to but also drive innovation in response to the evolving demands on healthcare systems, as highlighted in the introduction of this paper.

Institutional changes and new models are also being introduced as researchers explore opportunities for innovation in HE. It has been established that top management’s emphasis on knowledge value and the implementation of knowledge-based rewards positively impact the speed and quality of innovation within HE institutions [17]. Additionally, the rise in digitalisation and online learning represents a shift in education. Distance learning has been established for some time, and the use of hybrid learning and e-learning has increased [18]. These developments benefit students by providing flexibility and allowing them to manage responsibilities while studying. Institutions benefit by expanding course offerings and improving student access to required courses. García-Morales et al., 2021 assert that online learning is here to stay, with the COVID-19 pandemic contributing to its permanence [19]. These developments resonate with the broader context discussed in the introduction, where the need for innovative approaches in healthcare education is increasingly urgent.

## 4. Challenges of Innovation in Higher Education

### 4.1. Legacy Impacts of COVID-19

The COVID-19 pandemic had a profound influence on education worldwide. According to research conducted during this time, there was a rapid transformation in teaching methods and platforms to facilitate e-learning, as face-to-face learning was not feasible during certain phases of the pandemic [20]. Although remote learning possibilities were already established, Anderson et al. note that the pandemic led to a further decrease in classroom attendance, further highlighting the shift to online learning [21]. While this transition was crucial in the face of the pandemic, the shift towards online learning was likely inevitable even without COVID-19. Research has emphasised the need for adequate learning opportunities in preparation for potential future pandemics [22]. Despite the swift adaptation required, the education sector encountered many challenges in integrating online learning, revealing that it was not fully equipped to handle this sudden shift.

These observations are particularly relevant to the challenges discussed in the introduction, where the integration of innovative practices within HE is recognised as essential for preparing healthcare professionals for a rapidly changing environment. The pandemic has underscored the importance of equipping students with the necessary skills to adapt to a future immersed in digital healthcare [22]. Amankwaa et al., 2022 reinforce this sentiment, stating that organisations’ innovation initiatives heavily depend on employee human capital and behaviour at work and therefore may present as an opportunity for healthcare [23]. As digitalisation continues to evolve, it is evident that e-learning must play a crucial role in health science HE. Education must be adapted to new methods of learning that align with the ongoing developments in healthcare.

### 4.2. Ensuring Relevant and Evidence-Based Pedagogy

One of the key challenges in incorporating new innovations into education is supporting teaching staff in developing and adapting their teaching methods. Amankwaa et al. describe how staff faced considerable pedagogical challenges due to the rapid adjustments necessitated by the COVID-19 pandemic [3]. Many educators had limited experience with e-learning prior to the pandemic [20,23], and face-to-face learning from experienced professionals remains a preferred method in some cases, suggesting that it will continue to be an important component of HE.

This discussion resonates with the introduction’s emphasis on the challenges of implementing innovative practices in educational settings. The concept of learning by doing, rooted in the work of early educational progressives like John Dewey, posits that students learn most effectively through hands-on, real-world experiences [24]. Despite the abundance of online educational resources, the absence of bedside teaching in health-related programmes has negatively impacted students’ direct interaction with patients, compromising the development of physical examination and non-technical skills [25]. Papapanou et al. also highlight this issue, arguing that the limitation of interpersonal contact in e-learning hinders the development of essential communication and empathy skills needed for patient interaction [22]. Another challenge in e-learning is the potential for ethical issues, such as unequal access to reliable internet, which can exacerbate educational disparities [20]. Despite these challenges, the importance of preparing students in health science HE for a digital work environment remains paramount, aligning with the broader aim of fostering innovation in HE health programmes.

The integration of simulation-based learning has already been implemented in education programmes. Notably, the International Nursing Association of Clinical Simulation and Learning (INACSL) published a glossary for best practice, including digital opportunities, as early as 2016. Recent advancements in artificial intelligence (AI) may have further expanded the potential for enhancing simulation-based learning in HE [26].

### 4.3. Collaboration and Interdisciplinary Research

Collaboration and interdisciplinary approaches are essential in modern healthcare education and practice, yet individual disciplines often operate in silos, hindering the integration of diverse perspectives and expertise. The process of interdisciplinary collaboration ensures comprehensive treatment by integrating various resources and addressing systemic barriers, while also advocating for policy changes that benefit public health, particularly for vulnerable populations [27]. Barriers to transdisciplinary collaboration include limited time, geographical distance, and established disciplinary ways of working, which hinder an interdisciplinary approach [28]. This is supported by Ju et al. who describes differences in expertise which, along with varying levels of commitment, are considered the biggest obstacles to successful collaboration [29,30]. Similarly, Leon and Lipuma highlighted barriers to interdisciplinary research, such as varying disciplinary terminology, methodological discrepancies, and a culture of siloed disciplines. In response to these challenges, the researchers proposed strategies like fostering environments that encourage interdisciplinary dialogue, developing a common language for interdisciplinary communication, and providing transdisciplinary training, concluding that effective communication can enhance collaboration and research results. This ensures that all stakeholders can contribute effectively to advancing healthcare outcomes while bridging the gap between varied academic and professional practices.

## 5. The Opportunity for Innovation

While the concepts of innovation and health professions are not new, the intersection of these two domains has witnessed a dramatic acceleration in the past two decades [31,32,33]. This growth is a direct response to increasingly complex global health challenges, including workforce shortages, multimorbidity, and demands for more agile care delivery models. To fully harness the potential of innovation, however, the skillsets and training of health professionals must also evolve.

A seminal report, the 2019 Topol Review, highlighted the skills and technologies that clinicians will require in the digital age and how technological advances can improve the flexibility and responsiveness of training [34]. For example, immersive technologies like virtual reality (VR) can improve learning retention and clinicians’ ability to conduct complex procedures. While there is no substitute for real-world clinical training involving patient interactions, innovations like VR or augmented reality may provide a reliable, convenient, and auditable solution for training undergraduates, upskilling newly qualified clinicians, and enabling more experienced staff to maintain or improve their clinical skillsets [31].

In this dynamic and ever-changing healthcare landscape, allied health education must evolve. The integration of innovation, specifically in technology, digital health, and simulation, offers a promising avenue to enhance learning experiences and skill development for health professionals.

### 5.1. Moving from Traditional to Digital Approaches in HE

There is a growing body of evidence supporting the integration of digital innovations within health sciences higher education, one recent example being the use of virtual and high-fidelity simulation [23]. Ethical concerns regarding patient safety have become increasingly central in teaching and medical training, prompting the widespread adoption of virtual simulation as a standard component of training [35]. A systematic review with meta-analyses demonstrated that virtual simulation can enhance clinical reasoning in nursing students, although several factors need to be considered. For instance, 2D simulations appear to have greater impact compared to 3D, and longer sessions (lasting over 30 min) are associated with more substantial positive effects on clinical reasoning [36].

While practical training remains essential, institutions are developing strategies to mitigate the limitations of reduced face-to-face teaching. For instance, Imperial College London has created a digital library of patient encounters to mitigate the impact of reduced hands-on training resulting from e-learning [37]. Other digital formats are being explored to enrich educational delivery. Dedeilia et al. highlight the value of podcasts as tools for self-paced, in-depth learning [38]. Another innovative tool is Learning Analytics. As defined by the Society for Learning Analytics Research, this approach involves collecting, measuring, analysing, and reporting data on learners and their contexts. It aims to enhance the learning process and personalise the learning experience—tracing student profiles, abilities, and learning patterns to inform adaptive teaching strategies. This new and innovative approach, facilitated by digitalisation, presents important opportunities. Furthermore, research has shown that the introduction of student information systems and virtual learning environments such as Blackboard^®^ and Moodle^®^ has marked a shift in medical education. The e-learning era seems to open up great possibilities for collaboration between faculties and universities. Webinars can be utilised for interdisciplinary learning [39,40]. Lecture videos, online reading materials, etc., can be integrated into a “Massive Open Online Course”, which can be utilised either online or during classroom teaching [41,42,43]. These platforms not only support the delivery of educational content but also provide a foundation for broader pedagogical innovation and the integration of digital tools in healthcare higher education [44,45,46].

### 5.2. The Role of Organisation and Collaboration

As stated above many challenges exist for educators in the adoption digital transformation. This is also an organisational challenge that should be taken seriously. Gonsalo et al. state that to implement innovation in health systems, supportive structures are needed to manage its complexity [36]. Therefore, it should be argued that support from the institution and its stakeholders is crucial for innovation to happen. Amankwaa et al. observed that stakeholders can be inclined to make pedagogical adjustments without institutional support, highlighting a disconnect between faculty and administrators [3,23]. However, innovation within the higher education system involves a multitude of stakeholders beyond faculty, administrators, and government. Alumni and corporations can play a valuable role in the development of entrepreneurial, innovative campuses [14,47].

The financial shortfall is another organisational challenge that can hinder the progress of initiatives. Dula et al. advocate that although skills-based and in-person education is important, it requires substantial resource [48]. Underfunding issues are also being increasingly recognised, and it is proposed that education needs to focus on competencies, with interpersonal and trans-professional education requiring unified planning. Universities appear to be open to exploring funding models due to their endowment wealth [14]. Innovative funding opportunities are probably essential to empower innovation within the HE sector.

Furthermore, collaborations outside of the educational institutions should be further explored. Wells et al. report on a “Model of Collaborative Health Education, CPD [continuing professional development], Research and Innovation” [47,49]. This model allows healthcare employers to be involved in the development of education programmes. It is suggested that employer feedback is crucial in developing programmes and that a co-production model of collaboration between the health and social care sector and universities can have advantages to all parties involved, including professionalisation; skills and academic development; research and innovation; and engagement.

The integration of technology, digital health, and simulation into allied health education has the potential to impact the student learning experience. This impact is reflected in the UKPSF (UK Professional Standards Framework) Dimensions which emphasise the importance of understanding how students learn and how to use evidence-informed approaches to enhance student learning [39].

For example, evidence shows that the use of VR simulations in training improves student engagement and the retention of complex clinical skills [50]. However, it is essential to critically assess the long-term impact of such innovations on clinical competence and patient outcomes as well as respecting the individual learning styles which may not suit everyone. This requires a robust analysis of data related to academic practice, as outlined in the UKPSF. By systematically collecting and analysing data on student performance, satisfaction, and post-graduation outcomes, there is an opportunity to demonstrate the impact of these innovations on the student learning experience and make informed adjustments to the curriculum [39].

Simulation-based education has emerged as a cornerstone of allied health training, providing a safe and controlled environment for skill development and error analysis. High-fidelity simulators have become increasingly sophisticated, replicating real-world clinical scenarios with remarkable accuracy. However, the transferability of skills from simulation to clinical practice remains a subject of ongoing research and development. This also raises the question: is it the technology itself or the human factors that are most critical in enabling learning? Additionally, the cost-effectiveness of simulation compared to traditional training methods requires further exploration [50].

### 5.3. The Role of Entrepreneurship

The importance of institutional policies that promote innovation among students and staff has been recognised in the UK. While collaboration between large organisations and academia is challenging and requires time and effort from both sides, it also presents substantial opportunities [46]. The Scottish government’s strategy for entrepreneurial campuses highlights higher education as a key driver of innovation and economic development. This strategy emphasises the need to inspire young people to engage in entrepreneurial thinking, which necessitates adjustments to the curriculum, as well as leveraging extracurricular resources and external expertise [14].

The goal is that fostering an innovative and entrepreneurial approach within higher education can contribute to economic growth and development. According to Rubens et al. (2017) [38], universities are increasingly shifting from their traditional roles of teaching and research to embrace entrepreneurship, community engagement, and sustainable development [47,51]. These sustainable developments include not only economic growth but also contributions to environmental changes and social cohesion [52,53]. Research suggests that the presence of human capital—skills, knowledge, and expertise within a community—and institutional intellectual capital—collective knowledge, policies, and practices within institutions—correlates with increased economic growth and development [36]. Therefore, innovation in higher education could be a crucial factor in driving economic and societal progress.

### 5.4. Artificial Intelligence

The rapid growth and acceleration of AI present both substantial opportunities and potential barriers to innovation in health science higher education. AI is progressively reshaping how individuals engage, communicate, learn, and work. Chiu et al. describe the influence of AI on student learning, teaching, and assessment; in particular, the emergence of tools like ChatGPT has sparked widespread discussions, marking a substantive shift in public attitudes towards AI [22]. However, the deployment in education also raises concerns, including issues of data privacy, security, bias, and the evolving nature of teacher–student relationships [49]. Addressing these concerns is essential to ensure the responsible and ethical integration of AI in educational settings. It is not enough for students to merely become proficient in using AI; they must also understand that the nature of the work they are preparing for is undergoing changes due to its advancements. Universities have a responsibility to adequately prepare students for this shift, which requires a deep understanding of its implications both in education and within health science professions. By embracing these opportunities, higher education institutions can equip students with the necessary skills and knowledge to thrive in a rapidly evolving digital landscape [54]. AI is poised to revolutionise healthcare professions, enhancing efficiency and quality of care. These developments will shape the future roles of healthcare professionals and require new and adaptive competencies.

## 6. Challenges and Considerations

Despite the potential benefits of technological innovations in allied health education, several challenges must be addressed. The integration of technology into the curriculum requires substantial investment in infrastructure, faculty training, and ongoing support with linkage to pedagogy [55]. There are also concerns about the accessibility of technology for all students and those from diverse learning communities [56]. Moreover, the rapid pace of technological advancement necessitates continuous curriculum updates to ensure that students are learning relevant and contemporary skills [57]. This presents further technical challenges for staff to ensure that they understand and can adapt to the implications of quality assurance in particular within healthcare professional training and its regulation [23].

Another consideration is the potential impact of technology on the students’ expectations of healthcare and communication. While online learning and digital tools can facilitate independent learning, they may also lead to undesirable effects including a sense of isolation and a diminished capacity for empathic engagement—a key component of clinical practice [6,53,54]. It is crucial to maintain a balance between digital and face-to-face interactions to preserve the human element of education, with many aspects of care still face to face. Moving forward, ethical considerations also play a role in the adoption of technology in healthcare education [22]. The use of patient data in digital health tools raises questions about privacy, consent, and data security [31,32]. Finally, it is important to recognise that innovation is not synonymous with clinical improvement and/or patient outcomes; therefore, the core aims of healthcare must also be considered [55,56,57].

## 7. Conclusions

This narrative review has explored the multifaceted challenges and opportunities associated with fostering innovation in health science higher education. The increasing complexity of healthcare demands a dynamic and adaptive workforce, making it imperative for educational institutions to embrace interdisciplinary collaboration, innovative teaching methodologies, and new technologies. However, these technical advancements are not without their challenges, as traditional academic silos, varying regulatory environments, and the rapid pace of technological change can create barriers to effective implementation.

The role of entrepreneurship within HE is particularly crucial in driving innovation and economic development. By fostering an entrepreneurial mindset among students and staff, and by leveraging collaboration with external stakeholders, higher education institutions can become key contributors to societal progress. Furthermore, the integration of AI into education presents a transformative opportunity, albeit one that requires careful consideration of ethical implications and the evolving nature of professional roles.

As digital healthcare continues to evolve, it is crucial to equip students with the necessary skills to navigate and adapt to this transformation. The integration of technology in healthcare will not only reshape the roles of healthcare professionals but also demand new competencies in data management, telemedicine, and digital tools. By preparing students today, we ensure they are ready to meet the challenges and opportunities of a future driven by digital innovation, ultimately improving patient care and healthcare efficiency [22].

While new technologies and approaches can offer exciting possibilities, they must be rigorously evaluated to ensure that they are effective and beneficial in their own context. The adoption of innovation should be guided by evidence-based practices and a commitment to continuous improvement. The importance of considering the wider context of contemporary issues in higher education and how they impact teaching and assessment practices should also be emphasised.

Ultimately, for health science HE to respond to the demands of a rapidly changing world, it must continue to innovate and adapt. This will require not only the adoption of new technologies and pedagogies, but also the cultivation of a collaborative, interdisciplinary, and research-informed culture. Through this, higher education can play a pivotal role in preparing the next generation of healthcare professionals to meet future challenges with creativity, resilience, and a commitment to excellence.

## Data Availability

No new data were created.

## Public Involvement Statement

No public involvement in any aspect of this research.
